# Nucleolar protein NPM1 is essential for circovirus replication by binding to viral capsid

**DOI:** 10.1080/21505594.2020.1832366

**Published:** 2020-10-19

**Authors:** Jianwei Zhou, Yadong Dai, Cui Lin, Ying Zhang, Zixuan Feng, Weiren Dong, Yulan Jin, Yan Yan, Jiyong Zhou, Jinyan Gu

**Affiliations:** a MOA Key Laboratory of Animal Virology, Center of Veterinary Sciences, Zhejiang University, Hangzhou, Zhejiang, PR China; b Collaborative innovation center and State Key laboratory for Diagnosis and Treatment of Infectious Diseases, First Affiliated Hospital, Zhejiang University, Hangzhou, China

**Keywords:** Circovirus, viral capsid, nucleophosmin-1, nucleolar localization signal, nuclear entry

## Abstract

Entry of circovirus into the host cell nucleus is essential for viral replication during the early stage of infection. However, the mechanisms by which nucleolar shuttle proteins are used during viral replication is still not well understood. Here, we report a previously unidentified nucleolar localization signal in circovirus capsid protein (Cap), and that circovirus hijacks the nucleolar phosphoprotein nucleophosmin-1 (NPM1) to facilitate its replication. Colocalization analysis showed that NPM1 translocates from the nucleolus to the nucleoplasm and cytoplasm during viral infection. Coimmunoprecipitation and glutathione *S*-transferase pull-down assays showed that Cap interacts directly with NPM1. Binding domain mapping showed that the arginine-rich N-terminal motif ^1^MTYP**RRR**Y**RRRR**H**R**P**R**SHLG^20^ of Cap, and residue serine-48 of the N-terminal oligomerization domain of NPM1, are essential for the interaction. Virus rescue experiments showed that all arginine to alanine substitution in the N-terminal arginine-rich motif of Cap resulted in diminished viral replication. Knockdown of *NPM1* and substitution of serine-48 in NPM1 to glutamic acid also decreased viral replication. In addition, binding assays showed that the arginine-rich motif of Cap is a nucleolar localization signal. Taken together, our findings demonstrate that circovirus protein Cap is a nucleolus-located, and regulates viral replication by directly binding to NPM1.

## Introduction

Viruses are intracellular agents that rely on the host machinery for propagation [1]. Shuttle across the double-membrane nuclear envelope is essential for the replication of viruses [2–4]. Viruses have developed diverse strategies for nuclear shuttling. For example, herpes simplex virus type 1, adenovirus 2, hepatitis B virus, human immunodeficiency virus type 1 (HIV-1), and influenza A virus occupy the nuclear pore complex (NPC); adeno-associated virus and simian virus 40 may transit into the nucleus by disrupting the integrity of the nuclear membrane; reovirus type 3, vesicular stomatitis virus, poliovirus, Epstein-Barr virus, and Ebola virus might transit into the nucleus by altering the permeability of nuclear pores [5]. The capsid of herpes simplex virus type 1 and adenovirus 2, the nucleoprotein of influenza A virus, the Vpr, Rev and integrase of HIV-1, the core protein of hepatitis B virus, and NS5A of hepatitis C virus bind to NPC proteins (*i.e.*, nucleoporins), nuclear import soluble factors (*i.e*., importins), heat shock protein 70, and histone H1 [5–7].

*Circovirus*, a genus of the family *Circoviridae*, has been detected in terrestrial, aquatic and avian species, including pigs, ducks, dogs, minks, rats, palm civets, geese, pigeons, canaries, parrots, and others [8–15]. Circovirus is a nonenveloped, circular single-stranded DNA virus with a 1.7–2.0-kb genome [14,16]. Porcine circovirus types 2 (PCV2) and 3 (PCV3) have been demonstrated to be pathogenic to piglets, and the full genomes of PCV1, PCV2, PCV3, and PCV4 have been sequenced from pig herds [17,18]. PCV2, as the major causative agent of porcine circovirus-associated diseases, has been studied extensively. The viral structural proteins replicase (Rep) and capsid (Cap) were responsible for the rolling-circle replication of PCV DNA and the successive packaging of the PCV genome, and the nonstructural proteins ORF3 and ORF4 have been identified from PCV2-infected cells and tissues [19–25]. The Cap protein is essential for PCV2 replication and virus–host interaction. The Cap protein of PCV2 contains a nuclear localization signal (NLS), which consists of 41 amino acid residues at its N-terminus [26]. Circovirus trafficking depends on the cytoplasmic dynein IC1, which binds to Cap during the early stage of PCV2 infection [27], and conformational changes in Cap, but not virus disassembly, are critical for nuclear entry of circovirus [28]. However, the exact mechanism of nuclear shutting of circovirus during infection is still not well understood.

The nucleolar phosphoprotein nucleophosmin-1 (NPM1) takes part in diverse cellular processes such as ribosome biogenesis, DNA replication and repair, stress response, centrosome duplication, and nucleo–cytoplasmic transport [29]. NPM1, a nuclear shuttle factor, resides mainly in the nucleolus and consists of an N-terminal oligomerization domain (OligoD), a central histone-binding domain (HistonD), and a C-terminal nucleic acid-binding domain (NBD) [30,31]. NPM1 has been shown to interact with the Rev protein of HIV-1 [32] and adenoviral core proteins [33]. Proteomic data showed a slight upregulation of NPM1 expression in lymph nodes during PCV2 infection of piglet [34]. However, whether NPM1 is involved in PCV2 replication is not understood.

In the present study, we characterized the direct interaction of NPM1 with PCV2 Cap. Furthermore, silence of *NPM1* markedly inhibited PCV2 replication, indicating that NPM1 plays an important role in supporting PCV2 infection. In addition, virus rescue experiments showed that complete substitution of the arginine residues within the N-terminal arginine-rich motif (ARM) of Cap to alanine caused a significant reduction in PCV2 replication. To our knowledge, this is the first report of the identification of the Cap nucleolar localization signal of a circovirus, and of the role of NPM1 in replication.

## Materials and methods

### Cells and virus

PCV-free PK-15 cells were stored in our laboratory and maintained in minimal essential medium (Gibco, Carlsbad, CA, USA). HEK293T cells (CRL-11268; ATCC, Manassas, VA, USA) were cultured in Dulbecco’s modified Eagle’s medium (Gibco). All media were supplemented with 10% fetal bovine serum (CCS30010.02; MRC, Australia). PCV2 strain HZ0201 (accession no. AY188355), stored in our laboratory, was originally isolated from a pig with naturally occurring postweaning multisystemic wasting syndrome [35] and was propagated in PK-15 cells.

### Antibodies and reagents

Rabbit polyclonal antibodies (pAb) against Myc (R1208-1), GFP (SR48-02), FLAG (09^1^2-1), and mouse mAbs against β-actin (M1210-2) and GST (M0807-1) were purchased from Huaan Biological Technology (Hangzhou, China). Rabbit pAbs against histone H3 (R1105-1) and β-tubulin (0807-2) were also purchased from Huaan Biological Technology. Mouse anti-Myc (05-419) and anti-FLAG (F1804) mAbs were obtained from Sigma-Aldrich (St. Louis, MO, USA). Anti-FLAG affinity resin (A2220) for immunoprecipitation was also purchased from Sigma-Aldrich. Rabbit mAb against NPM1 (ab52644) was purchased from Abcam (Cambridge, MA). Mouse mAbs to Cap, Rep, ORF3, and ORF4 of PCV2 were produced by our laboratory [24,36–38]. NP-40 cell lysis buffer (50 mM Tris [pH 7.4], 150 mM NaCl, 1% NP-40) was purchased from Beyotime (P0013F; Shanghai, China). Horseradish peroxidase (HRP) or fluorescein isothiocyanate (FITC)-labeled goat anti-mouse and anti-rabbit IgG were purchased from KPL (Milford, MA, USA). Alexa Fluor 546-conjugated anti-mouse or anti-rabbit IgG were obtained from Invitrogen (USA).

### Treatment of cell cultures with chemical

Chemical used to pretreat PK-15 cells included the NPM1 inhibitor NSC348884 (S8149; Selleckchem, Houston, Texas, USA) (0.5 μM). Before treatment, cells were cultured overnight to 60% to 70% confluence and transferred to fresh medium in the absence or presence of the drug. The solvent dimethyl sulfoxide (DMSO) was used as a control.

### Plasmid construction and transfection

DNA fragments encoding full-length and truncated PCV2 *Cap* variants were amplified by PCR from full-length genomic DNA of PCV2 strain HZ0201, and subcloned separately into vectors pCMV-Myc-N (Clontech, Palo Alto, CA, USA), pCMV-FLAG-N (Clontech), pCMV-FLAG-N-GST (Clontech), pEGFP-C3 (Clontech), and pmCherry-C1 (Clontech) for different uses. The resultant plasmids were FLAG-Cap, FLAG-GST-Cap, Myc-Cap, mCherry-Cap, GFP-Cap, GFP-Cap(1-200aa), GFP-Cap(1-150aa), GFP-Cap(1-100aa), GFP-Cap(1-41aa), GFP-Cap(42-233aa), GFP-Cap(42-200aa), GFP-Cap(42-150aa), GFP-Cap(42-100aa), GFP-Cap(101-200aa), GFP-Cap(1-20aa), GFP-Cap(21-41aa). Plasmid Myc-ORF4 was constructed and stored in our laboratory [25]. The nucleotide fragment sumo-Cap was synthesized and inserted into the vector pET-28a (Novagen, Madison, WI) by Sunya Biotechnology (Zhejiang, China). The full-length cDNA sequences of *NCL* (accession no. XM_021074959.1), *NPM1* (accession no. XM_013990662.2), and truncated *NPM1* variants were amplified from PK-15 cells and cloned into vectors pmCherry-C1, pCMV-FLAG-N, and pEGFP-C3 and pGEX-4T-1 (GE Healthcare Biosciences, Piscataway, NJ, USA) using specific primers. The resultant plasmids were mCherry-NPM1, mCherry-NCL, FLAG-NPM1, GFP-NPM1(1-294aa), GFP-NPM1(1-117aa), GFP-NPM1(118-188aa), GFP-NPM1(189-294aa), GFP-NPM1(1-188aa), GFP-NPM1(118-294aa), and GFP-NPM1(1-117aa+189-294aa). Mutants were created by site-specific mutagenesis. Primers for cloning and quantitative real-time PCR are listed in Table 1. For transfection, PK-15 and HEK293T cells were seeded onto plates or glass coverslips at a suitable density according to the experimental scheme and grown to 70% to 90% confluence. jetPRIME transfection reagent (Polyplus Transfection, New York, NY, USA) was used for PK-15 cell transfection, and ExFect transfection reagent (T101-01/02; Vazyme Biotechnology, Nanjing, China) was used for HEK293T cell transfection according to the manufacturers’ instructions.

**Table 1.** Primers used for cloning and quantitative real-time PCR

Gene product	Sense primer (5ʹ to 3ʹ)	Anti-sense primer (5ʹ to 3ʹ)
Cap(1-233aa)	ATGACGTATCCAAGGAGGC	TTAAGGGTTAAGTGGGGGGTCT
Cap(1-200aa)	ATGACGTATCCAAGGAGGC	AGTGCCGAGGCCTACGTGGTCCACA
Cap(1-150aa)	ATGACGTATCCAAGGAGGC	TATGGTATGGCGGGAGGAGTAGTTT
Cap(1-100aa)	ATGACGTATCCAAGGAGGC	CTTTCTTATTCTGTAGTATTCAAAG
Cap(1-41aa)	ATGACGTATCCAAGGAGGC	TTTCCTTCTCCAGCGGTAACGGTGG
Cap(1-20aa)	AGCCATCTTGGCTAAGGATCCACCGGATCTAGATAACTGA	TCAGTTATCTAGATCCGGTGGATCCTTAGCCAAGATGGCT
Cap(21-41aa)	AATTCTGCAGTCGACGATGCAGATCCTCCGCC	GGCGGAGGATCTGCATCGTCGACTGCAGAATT
Cap(42-233aa)	AATGGCATCTTCAACAC	TTAAGGGTTAAGTGGGGGGTCT
Cap(42-200aa)	AATGGCATCTTCAACAC	AGTGCCGAGGCCTACGTGGTCCACA
Cap(42-150aa)	AATGGCATCTTCAACAC	TATGGTATGGCGGGAGGAGTAGTTT
Cap(42-100aa)	AATGGCATCTTCAACAC	CTTTCTTATTCTGTAGTATTCAAAG
Cap(101-200aa)	GTTAAGGTTGAATTCTGGCCCTGCT	AGTGCCGAGGCCTACGTGGTCCACA
NCL(1-724aa)	ATGGTAAAGCTCGCAAAGGCC	CTATTCAAACTTGGTCTTCTTTCCT
NPM1(1-294aa)	ATGGAAGATTCGATGGATAT	TTAAAGAGACTTCCTCCACT
mNPM1	TTTAGGGGCTGGCGCAAAAGACGAATTACATATTGTAGAAGCAGAGGCAATGAATTAT	ATAATTCATTGCCTCTGCTTCTACAATATGTAATTCGTCTTTTGCGCCAGCCCCTAAA
NPM1(1-117aa)	ATGGAAGATTCGATGGATAT	TACTAAGTGCTGTCCACTAATATGC
NPM1(118-188aa)	GCTGTAGAGGAAGATGCAGAGT	TTCCGCTTCCTCATCATCAAA
NPM1(189-294aa)	GAAAAAGCTCCAGTAAAGAAAT	TTAAAGAGACTTCCTCCACT
NPM1(1-188aa)	ATGGAAGATTCGATGGATAT	TTCCGCTTCCTCATCATCAAA
NPM1(118-294aa)	GCTGTAGAGGAAGATGCAGAGT	TTAAAGAGACTTCCTCCACT
NPM1(1-117aa+189-294aa)	ATGGAAGATTCGATGGATAT	CTTTACTGGAGCTTTTTCTACTAAGTGCTGTCCACTA
	TAGTGGACAGCACTTAGTAGAAAAAGCTCCAGTAAAG	TTAAAGAGACTTCCTCCACT
	ATGGAAGATTCGATGGATAT	TTAAAGAGACTTCCTCCACT
RT-NPM1	CGGTTGTGAACTAAAGGCCG	GAAACCGTCGGCTGTACAGA
GAPDH	TGGTGAAGGTCGGAGTGAAC	GGAAGATGGTGATGGGATTTC

### RT-qPCR

PK-15 cells were infected with PCV2 at an MOI of 1.0 or 5.0 for 0, 24, 48, and 72 h, and whole cell lysates were collected. Total cellular RNA was isolated using TRIzol reagent (Invitrogen) according to the manufacturer’s instructions. DNase I (M0303; NEB) was used to remove DNA. Reverse transcription of 1 μg of total RNA was performed using a RevertAid RT reverse transcription kit (K1691; Thermo Fisher, USA). The relative abundance of transcripts was assayed using ChamQ Universal SYBR qPCR master mix (Q711-02/03; Vazyme Biotechnology), the LightCycler 96 sequence detector system (Roche, Switzerland), and specific primers for *NPM1* and *GAPDH*.

### IFA and confocal microscopy

For IFA, PK-15 cells were mixed with PCV2 and seeded in 96-well flat-bottom microtiter plates at 100 μl/well. After incubation at 37°C for 72 h, cells were washed twice with phosphate-buffered saline (PBS) and fixed with a methanol–acetone mixture (1:1 [vol/vol]) at −20°C for 20 min. The cells were blocked with PBS containing 5% skim milk at 37°C for 1.0 h and incubated with mouse anti-Cap mAb at 37°C overnight, followed by incubation with FITC-conjugated goat anti-mouse IgG at 37°C for 1.0 h. Virus titers were determined by observing infected cells under a fluorescence microscope and calculating the TCID_50_ per 0.1 ml.

Confocal microscopy was used to assess colocalization of proteins. To observe the colocalization of Cap, Rep, ORF3, and ORF4 with NPM1, PK-15 cells were inoculated with PCV2 or transfected with Myc-ORF4. Cells grown on glass coverslips were fixed using 4% paraformaldehyde for 20 min and permeabilized with 0.2% Triton X-100 for 5 min at room temperature, then incubated at 4°C with primary antibodies overnight. The primary antibodies used included rabbit anti-NPM1 and mouse anti-Cap mAbs. After three washes with PBS containing 0.05% Tween 20, the cells were then incubated with FITC-labeled goat anti-mouse and Alexa Fluor 546-labeled donkey anti-rabbit IgG at 37°C for 1.0 h. To test the colocalization of NPM1, NCL and full-length PCV2 Cap or truncated Cap variants, HEK293T cells were cotransfected with indicated vectors fused with GFP and mCherry tags and then fixed using 4% paraformaldehyde for 20 min and permeabilized with 0.2% Triton X-100 for 5 min at room temperature. Cellular nuclei were then stained with 10 μg/ml 4ʹ, 6ʹ-diamidino-2-phenylindole (DAPI; 10236276001; Roche, Mannheim, Germany). The cells were then observed under an LSM780 laser scanning confocal microscope (Zeiss, Oberkochen, Germany). FITC fluorescence was detected after excitation at 488 nm with an emission long-band filter at 505 to 530 nm (green). Alexa Fluor 546 fluorescence was detected after excitation at 561 nm with an emission long-pass filter at 550 to 600 nm (red). DAPI was detected after excitation at 405 nm with an emission long-pass filter at 445 to 450 nm (blue).

### SDS-PAGE and immunoblotting

For western blotting, cells were lysed in lysis buffer after infection or other treatments. Lysates were collected, and proteins were separated by standard SDS-PAGE and transferred to nitrocellulose membranes (GE Healthcare) followed by blocking in PBS containing 5% skim milk and 0.05% Tween 20 and washing with PBS containing 0.05% Tween 20. The membranes were incubated with primary antibody overnight at 4°C. After three to five washes with PBS containing 0.05% Tween 20, the membranes were incubated with HRP-labeled secondary antibody at room temperature for 1.0 h. Immunoreactive protein bands were then visualized using enhanced chemiluminescence (Amersham Biosciences, Little Chalfont, United Kingdom) and imaged using AI680 Images (GE Healthcare), and protein band densities were normalized against the β-actin signal and quantified using ImageJ software.

### Co-IP and GST pull-down assays

For Co-IP assays, HEK293T cells were transfected with the indicated plasmids for 48 h. Cells were lysed in NP-40 cell lysis buffer containing a protease inhibitor cocktail. After centrifugation at 12,000 × *g* for 10 min, the supernatant was treated with protein A/G plus agarose (sc-2002; Santa Cruz Biotechnology, Santa Cruz, CA, USA) for 1.0 h at 4°C to eliminate nonspecific binding to the agarose gel, and immunoprecipitated using anti-FLAG beads. The beads were washed three times with NP-40 buffer and then boiled in protein loading buffer before the proteins were separated and subjected to western blotting. For the GST pull-down assays, His-sumo-Cap, GST, and GST-NPM1 were separately expressed in *Escherichia coli* BL21 cells. His-sumo-Cap was purified using Ni-NTA agarose (30210; QIAGEN, Germany). GST and GST-NPM1 were purified using Pierce glutathione agarose (21516; Thermo, Rockford, IL, USA). To prepare the bait proteins, purified GST, GST-NPM1 were immobilized on glutathione agarose beads, while His-sumo-Cap was used as the prey protein. A total of 500 ng of His-sumo-Cap was separately added to the GST, GST-NPM1 proteins and incubated overnight at 4°C and then washed with NP-40 lysis buffer and boiled with sample loading buffer. Finally, the samples were subjected to 12% SDS-PAGE and immunoblotted with anti-His and anti-GST mAbs. Moreover, for the unconventional GST pull-down assays, HEK293T cells were transfected with the indicated plasmids for 48 h. Cells were lysed in NP-40 buffer containing a protease inhibitor cocktail. Clarified buffer lysates were precleared and immobilized on glutathione agarose resin (16100; Thermo Fisher, USA) and incubated for 4 h at 4°C. The resin was washed three times with NP-40 buffer and then boiled in protein loading buffer before the proteins were separated and subjected to western blotting using mouse mAbs against FLAG and GST or rabbit pAb against GFP.

### *NPM1* knockdown by lentivirus-mediated RNA interference

*NPM1* knockdown was performed as previously described [25] with slight modifications. Briefly, four pairs of shRNAs targeting *NPM1* (shNPM1-1, −2, −3, and −4) and a negative-control shRNA (shCON) were designed using siRNA design software and then cloned into the lentivector pGreenPuro shRNA (SI505A-1; System Biosciences, Palo Alto, CA, USA). The shRNAs were transfected into PK-15 cells for screening of effective shNPM1 shRNAs. The effective shNPM1 (targeting sequence GGATGAGTTGCACATTGTATT) or shCON (targeting sequence CGGATCGCTACAAATAAG) RNA was co-transfected with the helper lentiviral packaging plasmids psPAX2.0 (12260; Addgene) and pMD2.G (12259; Addgene) into 293T cells to produce a lentivirus containing shNPM1 or shCON respectively. PK-15 cells were then infected with the resultant lentiviruses and cultured in complete medium for another 24 h and subjected to puromycin (5 μg/ml); A1113803; Invitrogen) treatment for 1 week to obtain *NPM1*-silenced cells. Finally, the viability of PK-15 cells expressing *NPM1* shRNA was determined using Cell Counting Kit-8 assays (C0037; Beyotime). To further analyze the function of NPM1 in PCV2 infection, *NPM1*-knockdown PK-15 cells or control cells were infected with PCV2 at an MOI of 1 and cultured. Cells were freeze-thawed three times and centrifuged at 12,000 × *g* for 10 min. Viral titers and protein expression levels were determined in PK-15 cells, and TCID_50_ values were calculated by the Reed-Muench method [27].

### Cellular fractionation

Isolation of nuclear and cytoplasmic components was performed using a nuclear and cytoplasmic protein extraction kit (P0027; Beyotime) as previously described [25]. Briefly, PCV2-infected PK-15 cells were treated with 200 μl of cytoplasmic protein extraction buffer A containing complete protease inhibitor cocktail for 5 to 10 min on ice, and then 10 μl buffer B were added. After vigorous vortex mixing and centrifugation at 12,000 × *g*, the nuclear-debris pellet was resuspended in 50 μl nuclear protein extraction buffer for 30 min and centrifuged for 5 min. SDS-PAGE and western blotting analysis were performed on the supernatant with mouse mAb to Cap and rabbit pAbs against histone H3 (a nuclear marker) and β-tubulin (a cytoplasmic marker).

### Generation of mutant PCV2

The mutant PCV2 (mA-PCV2) with residues Arg-5, −6, −7, −9, −10, −11, −12, −14 and −16 being replaced by alanines were cloned into vector pUC-19 to construct recombinant plasmids. The plasmids were sequenced to confirm that no errors were introduced as a result of PCR amplification and were then digested with *EcoR*I and self-ligated to produce the target PCV2 genes. Following that, the genome was transfected into PK-15 cells for continuous culture for 48 h, and once monolayer cells were grown, we performed serial passage of the PK-15 cells fifteen times to rescue viruses. Different passages (5, 10, and 15) of the rescued wt-PCV2 and mA-PCV2 were collected for PCV2 genome sequencing, and no nucleotide mutation was found in these PCV2 genomes, indicating the genetic stability of the rescued PCV2 during continued passage in cultured PK-15 cells.

### Virus growth curve

PK-15 cells were respectively infected with wt-PCV2 and mA-PCV2 at an MOI of 1.0, and collected at various time points. The samples were freeze-thawed three times and then centrifuged to collect the supernatant fraction. The TCID_50_ was calculated to draw viral growth curves.

### Statistical analysis

All results are presented as means ± standard deviations (SD). Statistical analysis was performed using Student’s *t-*test. *p-*values of < 0.05 were considered significant.

## Results

### NPM1 expression is essential for supporting PCV2 replication

**Figure 1.**
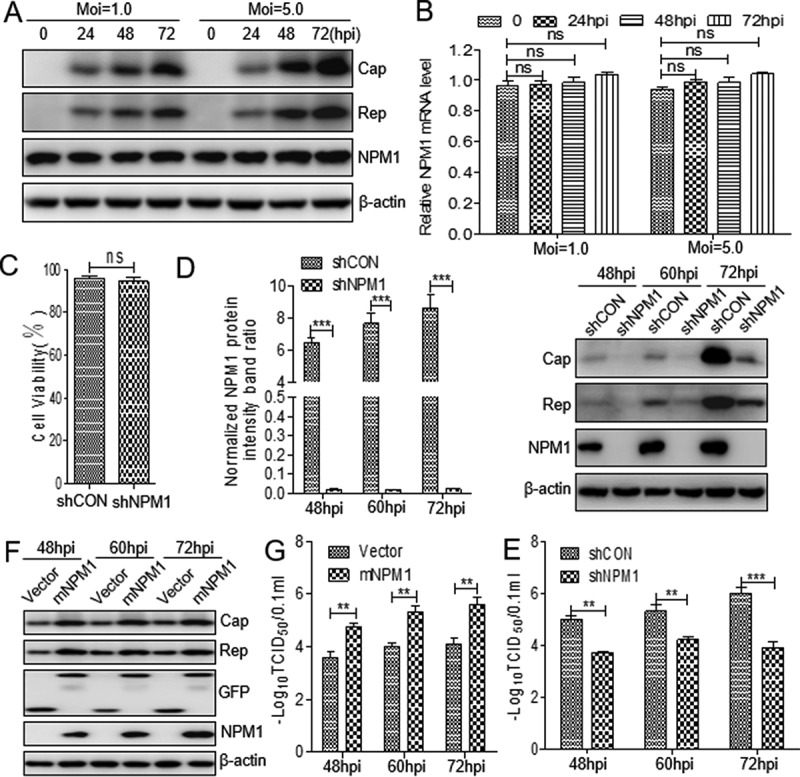
Nucleolar phosphoprotein nucleophosmin-1 (NPM1) expression sustained porcine circovirus type 2 (PCV2) replication. A. PK-15 cells were infected with PCV2 at an MOI of 1.0 or 5.0 for 0, 24, 48, and 72 h, and whole cell lysates were collected, and then analyzed by immunoblotting with rabbit anti-NPM1, mouse anti-capsid protein (Cap), mouse anti-replicase (Rep), and mouse anti-β-actin monoclonal antibodies (mAbs). B. Real-time quantitative PCR measurements of the *NPM1* mRNA abundance at the indicated times and MOIs described in panel A. Expression was normalized to the *GAPDH* mRNA level. C. The viability of PK-15 cells stably expressing a short hairpin RNA (shNPM1) was analyzed with a cell counting kit-8 assay. D. *NPM1*-silenced cells were infected with PCV2 at an MOI of 1.0 for 48, 60, and 72 h. The cell lysates were analyzed by immunoblotting to examine protein levels of Cap, Rep, NPM1 and β-actin. shCON-transfected cells were used as negative controls. E. The samples from D were used to measure PCV2 replication by determining TCID_50_ values. F. *NPM1*-silenced PK-15 cells were transfected with empty vector or GFP-mNPM1 for 24 h, respectively, and then infected with PCV2 at an MOI of 1.0 for 48, 60, and 72 h. The protein levels of Cap, Rep, NPM1 and β-actin were then determined by western blotting with the corresponding antibodies. G. The samples from F were used to measure PCV2 replication by determining TCID_50_ values. β-actin was used as a loading control. The NPM1 protein band intensity was analyzed using ImageJ software. Data are presented as means ± SD of three independent biological experiments. ns, not significant; **P < 0.01; ***P < 0.001

Proteomics data showed that NPM1 expression was slightly upregulated in lymph nodes of PCV2-infected piglet [34]. To validate these data, PK-15 cells were infected with PCV2 at a multiplicity of infection (MOI) of 1.0 and 5.0. Western blotting assay did not indicate an obvious change of NPM1 expression in PCV2-infected cells compared with mock-infected cells (Figure 1(a)). A similar result was observed for the level of the mRNA transcript of *NPM1* in PCV2-infected cells in RT-qPCR assay (Figure 1(b)). Subsequently, we constructed *NPM1-*silenced PK-15 cells to detect the level of PCV2 replication in the presence and absence of NPM1. Four pairs of short hairpin RNAs (shRNAs) specific for NPM1 were designed and synthesized to knockdown NPM1. These shRNAs were transfected into PK-15 cells by lentivirus-mediated shRNA transfer to establish cells stably expressing GFP (green fluorescent protein)-shRNAs. As shown in Figure 1(c), the viability of *NPM1*-silenced cells was similar to that of the shRNA control cells. Afterwards, these cells were inoculated with PCV2 at an MOI of 1.0, and viral replication was monitored by determining virus titer and the expression level of Cap and Rep proteins. The data showed that the expression of Cap and Rep were significantly inhibited in *NPM1*-silenced cells compared with shRNA control cells (Figure 1(d); *p* < 0.001). Similarly, the viral titer of PCV2 was also decreased significantly in *NPM1*-silenced cells (Figure 1(e); *p* < 0.01), suggesting that PCV2 replication is downregulated in *NPM1*-silenced cells.

Given that silencing NPM1 expression could inhibit PCV2 replication, we wanted to know whether transient overexpression of NPM1 could rescue the reduced levels of PCV2 replication in *NPM1*-silenced PK-15 cells. Since the shRNA targeting the coding region of NPM1, it may interfere with the expression of NPM1 plasmid, and we re-constructed a novel NPM1 non-sense mutant (mNPM1) and transfected the plasmid into the *NPM1*-silenced cells to sustain the NPM1 overexpression. The results showed that the expression of PCV2 Cap and Rep was markedly increased in *NPM1*-silenced cells transfected with mNPM1 during infection compared with cells transfected with empty vector (Figure 1(f)). Consistently, the production of PCV2 progeny virus was also significantly increased in *NPM1*-silenced cells transfected with mNPM1 compared with cells transfected with empty vector, suggesting that restoring NPM1 expression could revive reduced PCV2 replication (Figure 1(g)). Collectively, these results demonstrate that NPM1 supports PCV2 replication.

### The circovirus shuttles between the nucleolus and cytoplasm by binding to NPM1

**Figure 2.**
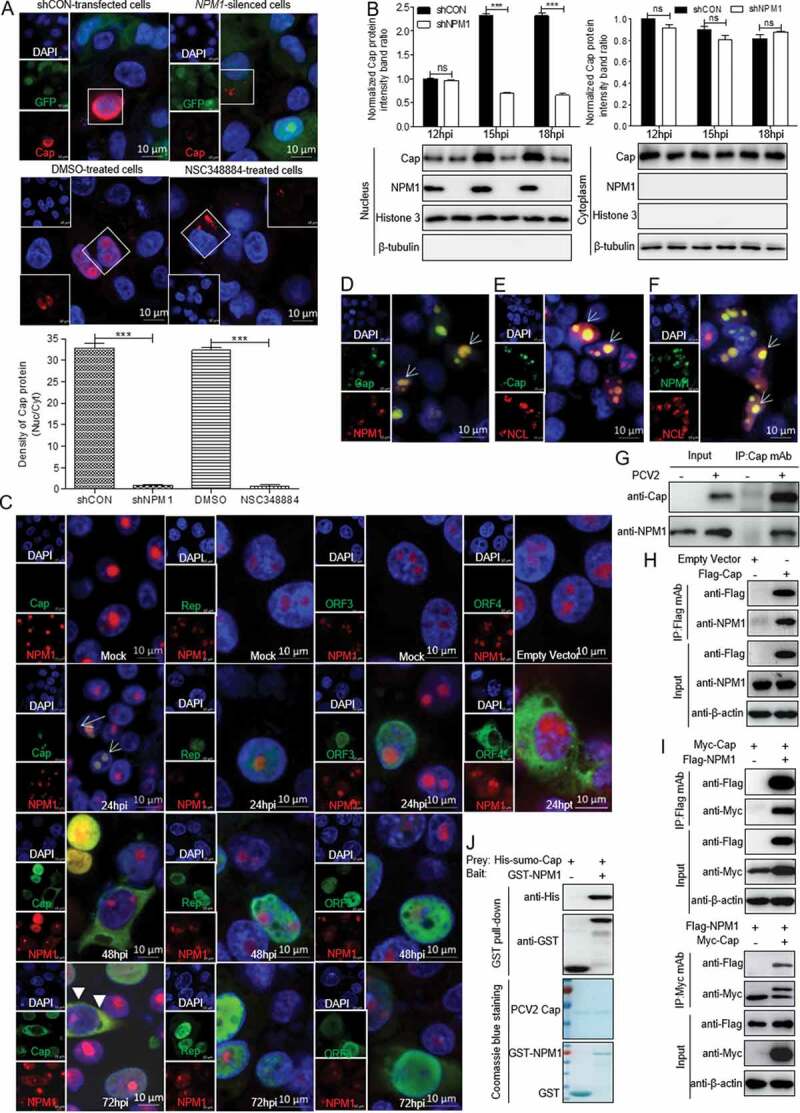
Direct interaction between NPM1 and Cap. A. *NPM1*-knocked down and shCON-transfected or 0.5 μM NSC348884- and DMSO-treated PK-15 cells were inoculated with PCV2 at an MOI of 25.0 for 15 h. Confocal microscopy analysis was performed with anti-Cap mAb as the primary antibody and Alexa Fluor 546-conjugated donkey anti-mouse antibody as the secondary antibody. GFP signals indicate silenced PK-15 cells. B. *NPM1*-knocked down PK-15 cells were infected with PCV2 at an MOI of 25.0 for 12, 15, and 18 h. shCON-transfected PK-15 cells were used as controls. The levels of Cap and NPM1 protein in the nucleus and cytoplasm were detected by immunoblotting with anti-Cap, anti-NPM1, anti-histone H3 (nuclear marker), and anti-β-tubulin (cytoplasmic marker) antibodies. The Cap and NPM1 protein band intensities or the intensities of nuclear/cytoplasmic resident viral protein Cap in the PCV2-infected shCON and shNPM1 or DMSO- and NSC348884-treated cells were analyzed using ImageJ software. Data are presented as means ± SD of three independent biological experiments. ns, not significant; ***P < 0.001. C. The colocalization of viral proteins Cap, Rep, ORF3, and ORF4 with NPM1. PK-15 cells were infected with PCV2 at an MOI of 1.0 for 24, 48, and 72 h or transfected with Myc-ORF4 for 24 h. The cells were fixed and stained with mouse mAbs against Cap, Rep, ORF3, and ORF4, rabbit anti-NPM1 antibody, FITC-labeled goat anti-mouse IgG, and Alexa Fluor 546-conjugated donkey anti-rabbit IgG. D. NPM1 colocalization with PCV2 Cap in transfected cells. HEK293T cells were cotransfected with GFP-Cap and mCherry-NPM1 for 24 h, and cells were fixed and then subjected to confocal observation. E and F. Nucleolin colocalization with Cap or NPM1 in transfected cells. HEK293T cells were cotransfected with mCherry-NCL and GFP-Cap (E) or GFP-NPM1 (F) for 24 h, and cells were fixed and then subjected to confocal observation. Nuclei were stained with 4ʹ, 6ʹ-diamidino-2-phenylindole (DAPI) (A, C-F). Scale bar, 10 μm. G. PK-15 cells were infected with PCV2 at an MOI of 1.0 for 48 h and cell lysates were immunoprecipitated with purified anti-Cap IgG mAb, followed by immunoblotting using corresponding antibodies. H. PK-15 cells were transfected with empty vector, and FLAG-Cap for 48 h. I. HEK293T cells were cotransfected with Myc-Cap and FLAG-NPM1 for 48h. The cell lysates were immunoprecipitated with FLAG beads (H, I) or anti-Myc purified IgG (I). J. Purified His-sumo-Cap separately mixed with the GST, GST-NPM1 proteins were pulled down with glutathione *S*-transferase (GST) beads, and then subjected to GST pull-down assays and immunoblotted with corresponding antibodies

NPM1 acts as a nuclear shuttle factor [30,31]. Whether NPM1 binds to a PCV2-encoded protein is still unclear. To investigate the role of NPM1 in the process of PCV2 nuclear entry, we observed the intracellular localization of viral protein Cap when *NPM1*-silenced and control cells or NSC348884- and DMSO- treated cells were inoculated with PCV2 for 12, 15, and 18 h. Confocal microscopy observations showed that at 15 h post-inoculation (hpi), Cap was mainly present in the cytoplasm in *NPM1*-silenced or NSC348884-treated cells, but it was present in the nucleus of control (shCON)-transfected or DMSO-treated cells (Figure 2(a)). Similarly, in western blotting assays, the level of Cap was significantly lower in the nucleus of *NPM1*-silenced cells at 15 and 18 hpi than in shCON-transfected cells (Figure 2(b), *p* < 0.001). These results suggest that inhibition of NPM1 expression blocks nuclear entry of PCV2 virions at an early stage of infection. Afterwards, wild-type PK-15 cells inoculated with PCV2 were cultured for 24, 48, and 72 h to determine the intracellular distribution of Cap and NPM1. Confocal microscopy images showed that the distribution of Cap overlapped with that of NPM1 in the nucleolus at 24 and 48 hpi, and in the cytoplasm at 72 hpi (Figure 2(c)). However, viral proteins Rep, ORF3, and ORF4 did not colocalize with NPM1 (Figure 2(c)). In addition, in cells cotransfected with GFP-Cap and mCherry-NPM1 or mCherry-nucleolin (NCL), we observed NPM1 colocalization with Cap or NCL and colocalization between Cap and NCL (Figures 2(d-f)). These data indicate that the NPM1 might serve as a nuclear transport receptor mediating nuclear entry of PCV2 during the viral life cycle.

To further explore the relationship between Cap and NPM1 in the process of nuclear shuttling, we detected the physical interaction between viral protein Cap and NPM1. Lysates of PCV2-infected or Cap-transfected PK-15 cells were immunoprecipitated with anti-Cap purified IgG or FLAG beads. In these coimmunoprecipitation (Co-IP) assays, NPM1 protein was readily detected in PCV2-infected or Cap-transfected PK-15 cells (Figures 2(g,h)), indicating that PCV2 Cap indeed interacted with endogenous NPM1 protein. To further study the interaction between PCV2 Cap and NPM1, 293T cells were cotransfected with FLAG-NPM1 and Myc-Cap, and then subjected to immunoprecipitation with FLAG beads or anti-Myc purified monoclonal antibody (mAb). Figure 2(i) showed that NPM1 interacted with PCV2 Cap. To investigate whether NPM1 bound directly to Cap, we performed glutathione *S*-transferase (GST) pull-down experiments. His-sumo-Cap together with GST and GST-NPM1 were respectively subjected to GST pull-down assays and immunoblotting. As shown in Figure 2(j), the products of GST-NPM1 could pull down His-sumo-Cap, but no pull-down was observed when empty vector was used. Taken together, these data indicate that PCV2 Cap interacts directly with NPM1.

### The N-terminal arginine-rich motif of Cap is a nucleolar localization signal and crucial for binding to NPM1

**Figure 3.**
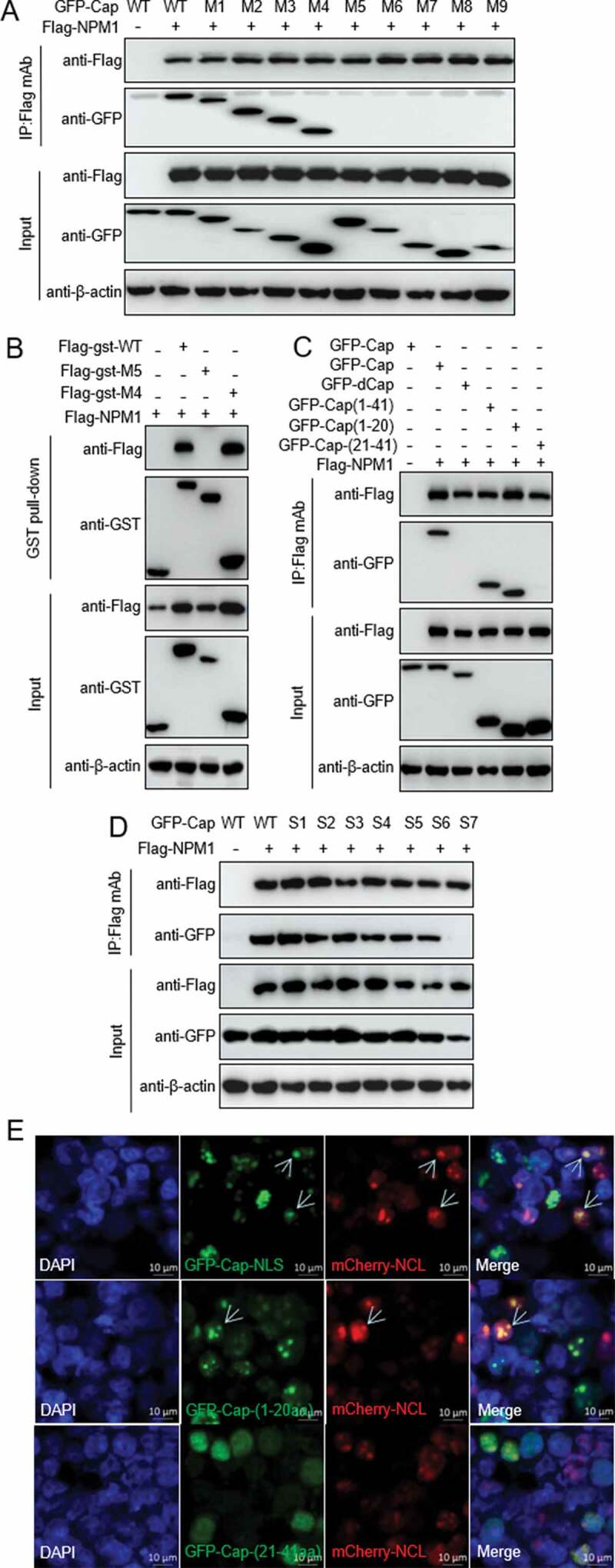
Identification of Cap binding domain to NPM1. A and B. The N-terminal nuclear localization signal of PCV2 Cap interacted with NPM1. HEK293T cells were cotransfected with plasmids encoding full-length PCV2 Cap or truncation mutants fused with a GFP-, or FLAG-GST tag, along with FLAG-NPM1; cell lysates were subjected to immunoprecipitation or GST pull-down and immunoblotting using the indicated antibodies. C. The Cap N-terminal amino acid residues 1 to 20 interacted with NPM1. HEK293T cells were cotransfected with plasmids GFP-Cap, GFP-dCap, GFP-Cap(1-41), GFP-Cap(1-20) and GFP-Cap(21-41), along with FLAG-NPM1. Cell lysates were immunoprecipitated with FLAG beads, followed by immunoblotting using the indicated antibodies. D. Identification of N-terminal arginine-rich motif of Cap responsible for the interaction between Cap and NPM1. HEK293T cells were cotransfected with Cap or Cap-S1, -S2, -S3, -S4, -S5, -S6, -S7 expression vectors, along with FLAG-NPM1, and the cell lysates were subjected to immunoprecipitation and immunoblotting using the indicated antibodies. E. Identification of the nucleolar localization signal in Cap. HEK293T cells were cotransfected with mCherry-NCL and GFP-Cap(1-41), GFP-Cap(1-20) or GFP-Cap(21-41) for 24 h. Then, the resultant cells were fixed, stained with DAPI and subjected to confocal observation. Scale bar, 10 μm

To identify the binding domain of PCV2 Cap responsible for the interaction with NPM1, we constructed various deletion mutants of PCV2 Cap. Co-IP assays showed that PCV2 Cap fragments consisting of amino acids (aa) 1-41 (M4), 1-100 (M3), 1-150 (M2), and 1-200 (M1) interacted with NPM1 (as did full-length Cap), whereas fragments consisting of aa 42-233 (M5), 42-200 (M6), 42-150 (M7), 42-100 (M8), and 101-200 (M9) did not interact with NPM1 (Figure 3(a)). GST pull-down assays indicated that PCV2 Cap-M4 interacted with NPM1, while Cap-M5 did not, suggesting that the N-terminal residues of PCV2 Cap (aa 1-41) are responsible for the interaction with NPM1 (Figure 3(b)). Further Co-IP experiments showed that the N-terminal peptide ^1^MTYPRRRYRRRRHRPRSHLG^20^ (domain I) of Cap, but not ^21^QILRRRPWLVHPRHRYRWRRK^41^, was responsible for the binding to NPM1 (Figure 3(c)). These data indicate that the N-terminal residues 1-20 of Cap that form an NLS are crucial for Cap binding to NPM1.

To identify the critical amino acid residues in this region that are responsible for binding to NPM1, we analyzed the primary sequence. Since NPM1 is an acidic protein, we screened for basic amino acid residues within the N-terminal domain I of NLS for PCV2 Cap and constructed seven arginine (R) to alanine (A) mutants: Cap-S1 (^5^AAA^7^), Cap-S2 (^9^AAAA^12^), Cap-S3 (^14^APA^16^), Cap-S4 (^5^AAAYAAAA^12^), Cap-S5 (^9^AAAAHAPA^16^), Cap-S6 (^5^AAAYRRRRHAPA^16^), and Cap-S7 (^5^AAAYAAAAHAPA^16^). These mutant constructs were cotransfected with FLAG-NPM1 into 293T cells. Wild-type (WT) Cap and all the mutants except Cap-S7 bound to the FLAG-NPM1 (Figure 3(d)). These results demonstrate that the ARM of N-terminal domain I of PCV2 Cap is required for binding to NPM1.

As shown in Figure 2(c), Cap is located in the nucleolus at the early stage of PCV2 infection. To investigate whether PCV2 Cap has a nucleolus localization signal (NoLS), we used online tools (NucleOlar location sequence Detector, http://www.compbio.dundee.ac.uk/www-nod/index.jsp; and NLS Mapper, http://nls-mapper.iab.keio.ac.jp/cgi-bin/NLS Mapper form.cgi). Surprisingly, a potential NoLS was predicted at the N-terminus of PCV2 Cap. To validate this, plasmids Cap-M4, GFP-Cap(21-41aa), and GFP-Cap(1-20aa), -S1, -S2, -S3, -S4, -S5, -S6 and -S7, were respectively cotransfected into 293T cells along with mCherry-NCL. Confocal imaging showed that only NLS and the N-terminal residues ^1^MTYPRRRYRRRRHRPRSHLG^20^ of NLS, as well as various ARM mutants except the Cap-S7 (data not shown) and C-terminal residues 21-41 of the NLS (Figure 3(e)), colocalized with NCL. These data confirm that the ARM of the N-terminal peptide ^1^MTYPRRRYRRRRHRPRSHLG^20^ within the N-terminus of PCV2 Cap is the NoLS.

### The binding of unphosphorylated serine-48 of NPM1 to Cap is key to regulating PCV2 replication

**Figure 4.**
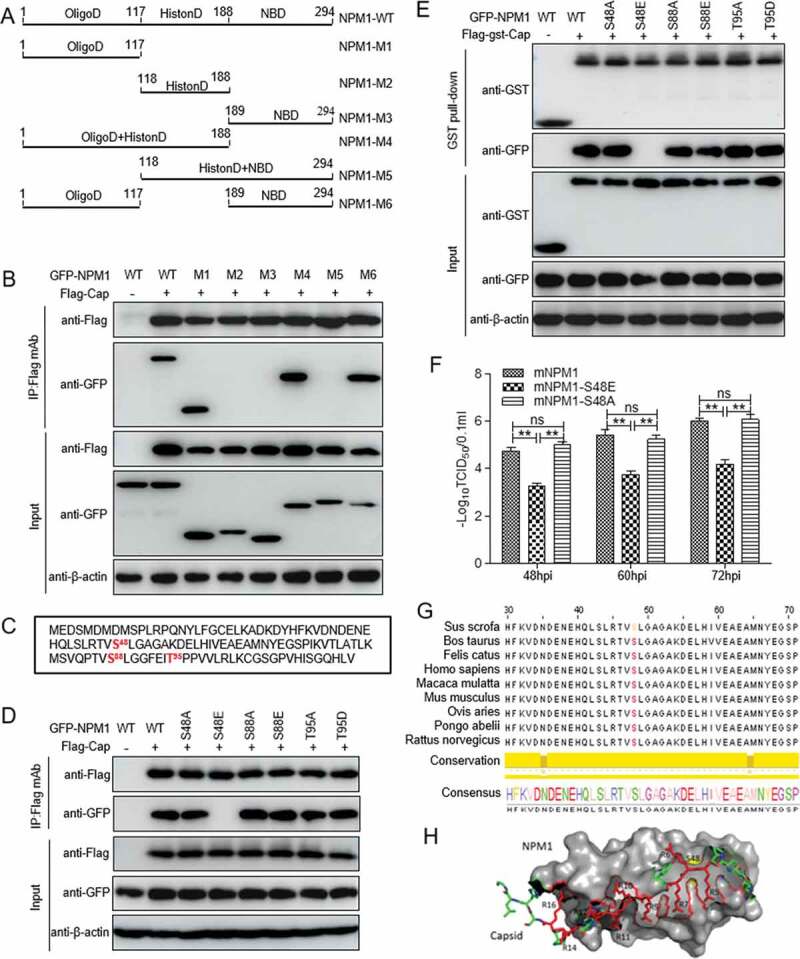
Unphosphorylated Serine 48 of NPM1 binds to Cap, regulating PCV2 replication. A. Schematic representation of the OligoD, HistonD, and nucleic acid-binding (NBD) domains of NPM1 and their truncation mutants used in this study. B. The OligoD domain of NPM1 (amino acids [aa] 1 to 117) interacted with PCV2 Cap. HEK293T cells were cotransfected with plasmid GFP-NPM1-WT or serial GFP-NPM1 truncated mutants M1 to M6, along with FLAG-Cap. The cell lysates were immunoprecipitated with FLAG beads and immunoblotted using the indicated antibodies. C. The N-terminal amino acid sequence of the OligoD domain of NPM1. Serine (S) or threonine (T) residues are marked in red. D and E. Mapping the crucial amino acids of OligoD responsible for binding to PCV2 Cap. HEK293T cells were cotransfected with NPM1 or NPM1 variants (-S48A, -S48E, -S88A, -S88E, -T95A, or -T95D), along with FLAG-Cap and FLAG-GST-Cap expression vectors, and the cell lysates were subjected to immunoprecipitation or GST pull-down and immunoblotting using the indicated antibodies. F. *NPM1*-silenced PK-15 cells were transfected with the indicated mNPM1 plasmids for 24 h and were infected with PCV2 at an MOI of 1.0 for 48, 60, and 72 h. Viral titers were then determined by TCID_50_. Data are presented as means ± SD of three independent biological experiments. ns, not significant; **P < 0.01. G. Alignment of amino acid residue Ser48 in NPM1 from different genera. H. Prediction of binding pattern of N-terminus of OligoD domain from NPM1 with N-terminal residues 1 to 20 of PCV2 Cap using PyMOL software

NPM1 contains several functional domains, such as OligoD (bearing chaperone activity), the C-terminal NBD, and central acidic domains for histone binding (HistonD) [31]. To identify the domain responsible for the binding of NPM1 to Cap protein, we constructed a series of NPM1-truncation mutants fused with GFP: OligoD (aa 1-117), HistonD (aa 118-188), NBD (aa 189-294), OligoD-HistonD (aa 1-188), HistonD-NBD (aa 118-294), and OligoD-NBD (aa 1-117+189-294) (Figure 4(a)). Co-IP assays showed that the constructs OligoD, OligoD-HistonD and OligoD-NBD interacted with PCV2 Cap, whereas HistonD, NBD and HistonD-NBD did not interact (Figure 4(b)), indicating that the OligoD domain of NPM1 is responsible for the interaction with PCV2 Cap.

Phosphorylation of the OligoD domain is essential for the regulation of NPM1 structural polymorphism and its nucleolar localization [39]. To investigate whether phosphorylated sites within OligoD are involved in the interaction between NPM1 and PCV2 Cap, we cotransfected plasmids GFP-NPM1-S(serine)48A(alanine), -S48E (glutamic acid), -S88A, -S88E, -T(threonine)95A, and -T95D(aspartic acid) into 293T cells with FLAG-Cap and FLAG-GST-Cap, respectively (Figure 4(c)). Intriguingly, the Co-IP and GST pull-down assays demonstrated that the non-phosphorylated S48A, S88A and T95A, and mimic-phosphorylated S88E and T95D bound to FLAG-Cap and FLAG-GST-Cap as well (or nearly as well) as the WT. However, the mimic-phosphorylated S48E variant lost the ability to bind to PCV2 Cap (Figures 4(d,e)). These findings indicate that unphosphorylated Ser48 within the OligoD domain of NPM1 is responsible for the interaction between NPM1 and the viral protein Cap. To assess whether Ser48 phosphorylation inhibits replication of PCV2 inhibition, *NPM1*-knocked down cells were transfected with plasmids expressing mNPM1 or its mNPM1-S48E or mNPM1-S48A variants, and infected with PCV2. The viral titer in *NPM1-*knocked down cells transfected with mNPM1-S48E was significantly reduced compared with that in cells transfected with mNPM1-S48A or mNPM1 (Figure 4(f); *p* < 0.01), demonstrating that Cap binding to unphosphorylated Ser48 within the OligoD domain of NPM1 is critical for PCV2 replication. We aligned sequences of the OligoD domain of NPM1 from various species using the Jalview software suite. Figure 4(g) shows that the Ser48 site of NPM1 OligoD is highly conserved. We also assessed the binding pattern of the OligoD domain of NPM1 with domain I of PCV2 Cap *in silico* using PyMOL software. The data in Figure 4(h) show that the N-terminal OligoD domain of NPM1 bound well to the N-terminal domain I of PCV2 Cap. Collectively, the data demonstrate that unphosphorylated Ser48 within the OligoD domain of NPM1 is critical for the binding of NPM1 to the viral protein Cap.

### The N-terminal ARM of Cap protein is important for PCV2 replication

**Figure 5.**
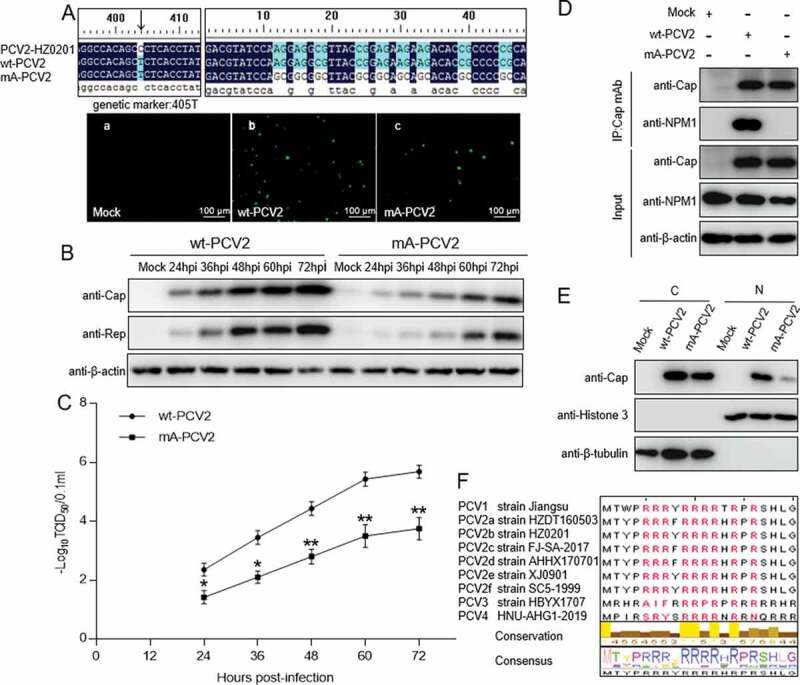
Rescue of PCV2 with arginine to alanine substitution in the nucleolar localization signal. A. wt-PCV2 and mA-PCV2 with residues Arg-5, −6, −7, −9, −10, −11, −12, −14 and −16 being replaced by alanines were rescued on PK-15 cell monolayers and rescue of wt-PCV2 and mA-PCV2 were validated by anti-Cap immunofluorescence assays and Sanger sequencing. (a) Mock-infected cells; (b) wt-PCV2-infected cells; (c) mA-PCV2-infected cells. Scale bar, 100 μm. B. Western blotting of viral proteins from wt-PCV2 and mA-PCV2. PK-15 cells were infected with wt-PCV2 or mA-PCV2 at an MOI of 1.0 and cultured for the indicated times. Cell lysates were subjected to western blotting with the indicated antibodies. β-actin was used as a loading control. C. One-step growth curves of wt-PCV2 and mA-PCV2. TCID_50_ was detected in wt-PCV2- or mA-PCV2-infected samples from panel B. Data are presented as means ± SD of three independent biological experiments. *P < 0.05; **P < 0.01. D. The Cap protein of mA-PCV2 lost the ability to bind to NPM1 in infected cells. PK-15 cells were infected with wt-PCV2 at an MOI of 1.0 or mA-PCV2 at an MOI of 5.0 for 48 h. Mock infected cells was used as controls. E. The levels of PCV2 Cap protein in the cytoplasm and nucleus of wt-PCV2- and mA-PCV2-infected cells. PK-15 cells were mock infected or infected with wt-PCV2 and mA-PCV2 at an MOI of 25.0 for 15 h, and the viral protein Cap levels in the cytoplasm and nucleus were separated using a nuclear and cytoplasmic extraction kit, and were detected by immunoblotting with anti-Cap, anti-histone H3 (nuclear marker), and anti-β-tubulin (cytoplasmic marker) antibodies. Data are presented as means ± SD of three independent biological experiments. C, cytoplasmic extracts; N, nuclear extracts. ***P < 0.001. F. Amino acid sequence alignment of the arginine-rich motifs from different PCV genotypes. Arginine residues are marked in red

As the results of experiments described above showed that the ARM in the N-terminus of the Cap protein is crucial for binding to NPM1, we aimed to investigate whether the ARM of Cap is also essential for PCV2 replication. Using PCV2 strain HZ0201 as a model, by direct mutation techniques, all arginine residues within the N-terminal ARM of Cap were replaced with alanine to generate an ARM-substituted PCV2 mutant (mA-PCV2). In parallel, as a control, cytosine^405^ within the *Cap* gene was nonsense-mutated to thymine to generate a form of PCV2 with a genetic marker (wt-PCV2). Using infectious clone techniques, the two mutants were respectively transfected into PK-15 cells. Immunofluorescence assays (IFA) showed detection of a Cap signal with anti-Cap mAb in PK-15 cell monolayers (Figure 5(a)). To assess the characteristics of mA-PCV2 virus, we analyzed their replication potential. In a western blotting assay, the expression levels of viral proteins Cap and Rep were significantly decreased in mA-PCV2-infected cells compared with wt-PCV2-infected cells (Figure 5(b); *p* < 0.001). Consistently, the production of virion progeny in mA-PCV2-infected cells was markedly decreased compared with that in wt-PCV2-infected PK-15 cells (Figure 5(c); *p* < 0.01). To investigate whether the lower replication ability of mA-PCV2 involved NPM1, we validated the interaction of NPM1 with Cap. In mA-PCV2-infected cells, Cap binding to NPM1 was not detectable, whereas it was clear in wt-PCV2-infected cells (Figure 5(d)). Similarly, western blotting assay showed a lower level of Cap protein expression in the nucleus of mA-PCV2-infected cells compared with wt-PCV2-infected cells (Figure 5(e)). Finally, we aligned the amino acid sequences of the Cap NoLS from different PCV genotypes; the ARM is identical in different genotypes of PCV2 (Figure 5(f)). Taken together, our data confirmed that the ARM of Cap is important for replication of PCV2.

## Discussion

NPM1, a versatile protein, interacts not only with multiple host cell factors, but also with different viral proteins. Because of its constitutive expression, intracellular dynamics, and binding capacities, NPM1 acts as a critical regulator of viral replication at different stages such as nuclear import, viral genome transcription and assembly, and particle formation [40–43]. In physiological conditions, NPM1 principally resides in the nucleolus, though it contains domains for cytoplasmic, nucleoplasmic, and nucleolar localization. Two stretches of negatively charged amino acids in the primary structure of NPM1 are important because these motifs may potentially bind with positively-charged amino acid stretches in viral proteins, such as an ARM or NLS [44]. In a recent report, NPM1 expression was found to be slightly upregulated in PCV2-infected inguinal lymph nodes of piglets [34]. In the present study, overexpression of NPM1 could revive the reduced levels of PCV2 replication in *NPM1*-silenced cells (Figures 1(f,g)). Therefore, we are interested in the role of NPM1 during PCV2 infection.

DNA viruses adopt multiple pathways to get into the nucleus, for example via delocalization of nucleolar proteins, or hijacking/modifying different cellular processes that occur in the nuclear region [5,45–47]. For instance, some viruses encode viral proteins containing an NLS to enter the nucleus via the nuclear pore complex; other viruses gain entry into the nucleus by disrupting the integrity of the nuclear membrane, or altering the permeability of nuclear pores [5]. Several viral proteins not only bind to NPC proteins (*i.e*., nucleoporins) or to nuclear import soluble factors (*i.e*., importins), but also interact with cellular proteins such as Hsp70 and histone H1 to pass through the NPC [5–7]. PCV2 is the smallest DNA virus that can replicate autonomously in the nucleus. To deliver its genome into the nucleus, intact capsid is transported through microtubules to reach the microtubule organizing centers [27,28]. Normally, the NLS of PCV2 Cap is buried inside the capsid and not exposed on the surface of mature virions, but it may become exposed in the metastable capsid during entry into host cells, named “viral breathing” [48]. In the present study, the N-terminal amino acid sequence ^1^MTYPRRRYRRRRHRPRSHLG^20^ of Cap was identified as a NoLS (Figure 3(e)), required for the entry of PCV2 virion into the nucleolus during infection. Interestingly, the NLS of Cap contains two positively charged amino acid segments, NLS-A and -B. NLS-A is an arginine-rich domain, and may function as a novel cell-penetrating peptide capable of entering into cells, while NLS-B does not [49].

The interaction of viral proteins with nucleolar components indicates an important role of the nucleolus in the life cycle of certain viruses, and may explain why viral proteins have been observed in the nucleolus. Viral exploitation of nucleolar function may lead to alterations in host cell transcription and translation, or to hijacking of nucleo–cytoplasmic transport to facilitate viral replication [50]. In the present study, we demonstrate that PCV2 Cap interacts directly with NPM1 (Figure 2(j)), and the ARM of the Cap NoLS is essential for the interaction (Figure 3(d)). When the ARM of the Cap NoLS was substituted, Cap lost the ability to bind to NPM1 and the replication ability of PCV2 was markedly reduced (Figures 5(b-d)). Intriguingly, once residue Ser48 of NPM1 was substituted to glutamic acid, NPM1 was distributed to the nucleoplasm (from the nucleolus), and did not bind to PCV2 Cap, resulting in diminished viral replication (Figures 4(d-f)). This suggests that residue Ser48 of NPM1 is crucial for its binding with PCV2 Cap, and ultimately for viral replication. Considering that sequential phosphorylation of solvent exposed Ser residues modulates the thermodynamic stability of the NPM1 oligomerization domain to promote exposure of other sites buried within the pentamer structure for subsequent phosphorylation and to drive the structural switch from pentamer to disordered monomer [39], one may hypothesize that the Ser48 substitution of NPM1 alters the spatial conformation of NPM1. Moreover, considering that the ARM at the N-terminus of PCV2 Cap functions as an NPM1 binding site and mediates PCV2 transport to the nuclear compartment, we hypothesize that NPM1 serves as an adaptor of PCV2 virion to facilitate the intracellular trafficking of circovirus, and hence it is essential for viral replication inside the nucleus of infected cells.

In conclusion, we identified that the arginine-rich N-terminal motif of PCV2 Cap binds directly with the N-terminal OligoD domain of NPM1 that can shuttle between the host cell nucleolus and the cytoplasm, to facilitate nuclear entry of PCV2 virion. Furthermore, the ARM within the NoLS of Cap, and Ser48 of NPM1, are crucial for PCV2 replication. These results not only increase our understanding of the mechanism of circovirus replication, but also identify a novel target for designing antiviral drugs and vaccine against circovirus.
